# Benefits of Aeronautical Preform Manufacturing through Arc-Directed Energy Deposition Manufacturing

**DOI:** 10.3390/ma16227177

**Published:** 2023-11-15

**Authors:** Alfredo Suárez, Pedro Ramiro, Fernando Veiga, Tomas Ballesteros, Pedro Villanueva

**Affiliations:** 1 Tecnalia, Basque Research and Technology Alliance (BRTA), Paseo Mikeletegi 7, 20009 Donostia-San Sebastian, Spain; alfredo.suarez@tecnalia.com (A.S.); pedro.ramiro@tecnalia.com (P.R.); 2 Engineering Department, Public University of Navarra, Los Pinos Building, Arrosadia Campus, 31006 Pamplona, Spain; tomas.ballesteros@unavarra.es (T.B.); pedro.villanueva@unavarra.es (P.V.)

**Keywords:** aircraft structures, wire arc additive manufacturing (WAAM), titanium Ti_6_Al_4_V preforms, near-net shape, material efficiency, sustainable manufacturing

## Abstract

The paper introduces an innovative aerospace component production approach employing Wire Arc Additive Manufacturing (WAAM) technology to fabricate near-finished preforms from Ti_6_Al_4_V titanium. Tensile tests on WAAM Ti_6_Al_4_V workpieces demonstrated reliable mechanical properties, albeit with identified anisotropic behavior in horizontal samples, underscoring the need for optimization. This alternative manufacturing strategy addresses the challenges associated with machining forged preforms, marked by a high Buy To Fly (BTF) ratio (>10), leading to material wastage, prolonged machining durations, elevated tool expenses, and heightened waste and energy consumption. Additionally, logistical and storage costs are increased due to extended delivery timelines, exacerbated by supply issues related to the current unstable situation. The utilization of WAAM significantly mitigates initial BTF, preform costs, waste production, machining durations, and associated expenditures, while notably reducing lead times from months to mere hours. The novelty in this study lies in the application of Wire Arc Additive Manufacturing (WAAM) technology for the fabrication of titanium aircraft components. This approach includes a unique height compensation strategy and the implementation of various deposition strategies, such as single-seam, overlapping, and oscillating.

## 1. Introduction

The recent surge in demand for titanium alloy components in the aerospace industry necessitates innovative manufacturing methods to enhance design flexibility, reduce environmental impact, and lower current manufacturing costs. Wire Arc Additive Manufacturing (WAAM) is emerging as a promising alternative to traditional subtractive methods. WAAM involves melting material using a heat source and depositing it layer by layer to form a preform closely resembling the final component [1]. Directed Energy Deposition (DED), a subset of WAAM, utilizes a coaxial feeder for material in wire or powder form and various energy sources for melting [2,3]. Utilizing wire as raw material offers cost reduction and higher material efficiency compared to powder. Hence, WAAM, whether employing gas metal arc welding (GMAW), tungsten inert gas welding (GTAW), or Plasma Arc Welding (PAW), proves to be a cost-effective and high contribution rate process for producing large aerospace parts of low to medium complexity [4,5,6].

In PAW, a plasma arc is formed between a non-consumable tungsten electrode and the substrate, with argon forced through the torch to create the plasma arc. This results in high energy density, enhancing arc stability, and reducing contamination compared to processes like GTAW [7]. Important studies have given a boost to the implementation of WAAM titanium technologies in aeronautical applications [8,9,10]. Many of these studies focus on the application of an interlayer hot rolling process [11,12] and interlayer dwell times [13,14] to improve the quality and applicability of the process.

In order to implement this technology, important research efforts have been made at all stages, from the design to the process itself and to the post-processing. Design for Additive Manufacturing (DfAM) in the context of metal components, especially using technologies like Wire Arc Additive Manufacturing (WAAM), is a strategic approach focusing on the unique opportunities and challenges presented by metal additive processes [15]. It involves crafting intricate geometries that leverage the design freedom inherent in AM, allowing for the optimization of shapes to enhance mechanical properties. Topology optimization is employed to generate structures that are not only lightweight but also robust [16].

In the realm of Wire Arc Additive Manufacturing (WAAM), the meticulous control of thermal dynamics and layer height is paramount for ensuring the efficacy of the fabrication process. Thermal control intricately manages parameters like arc voltage, current, and travel speed to regulate heat input during material deposition [17]. Striking the right balance is crucial to prevent issues like distortion or incomplete fusion. Simultaneously, maintaining a uniform layer height is pivotal for upholding the structural soundness and surface quality of the final component. Achieving this control involves precise movements of the print head or deposition tool, guaranteeing consistency in layering [18].

WAAM employs various strategic approaches in path deposition, each tailored to specific requirements [19]. The single-seam strategy, involving continuous material deposition, is efficient for building simpler geometries. On the other hand, the overlapping strategy enhances layer bonding, albeit with increased material usage. The oscillating strategy introduces controlled zigzag movements during deposition, contributing to improved surface finish and defect mitigation. The judicious selection of these path strategies depends on factors such as part geometry, material characteristics, and desired outcomes, underlining the need for optimization to harness WAAM’s potential for complex metal component fabrication [20].

In plasma spray technology, a sophisticated two-dimensional magnetohydrodynamic model has been developed for predicting the steady-state properties of an argon-operated plasma spray torch [21]. In ref. [22], a steady operating mode is simulated through the development of a two-dimensional and stationary magnetohydrodynamic model for a plasma spray torch using argon. This model aims to predict the plasma properties during operation. Additionally, a unified model explores various modes of direct current discharges in argon, while another self-consistent model for DC gas discharges in molecular gases sheds light on discharge modes and the relationship between voltage drop and current density [23]. These models enhance our understanding of complex phenomena in plasma spray and gas discharges.

Titanium and its alloys exhibit a high affinity for oxygen, nitrogen, and hydrogen, which increases with temperature. Oxygen and nitrogen, as alpha-phase stabilizers, anchor dislocations in deformation, affecting tensile behavior. Managing oxygen levels is crucial to optimize fracture toughness and tensile strength [24]. Regarding hydrogen, specifications often limit its content to a maximum of 125 to 200 ppm depending on the titanium alloy. Above these limits, hydrogen embrittles some titanium alloys, reducing impact and tensile strength and causing delayed cracking. An oxidizing atmosphere helps reduce hydrogen uptake by lowering its partial pressure and providing a protective oxide surface to titanium [18,25,26,27,28].

This paper explores the feasibility of utilizing Wire Arc Additive Manufacturing (WAAM) compared to other additive manufacturing (AM) technologies for titanium aerospace components, each exhibiting distinct characteristics [29]. WAAM, employing wire deposition, may yield lower material efficiency and require additional processing for a smoother surface. While excelling in the rapid production of large components, it might be less suitable for intricate geometries. In contrast, Powder Bed Fusion (PBF) methods [30], like Selective Laser Melting (SLM) [31] or Electron Beam Melting (EBM) [32], provide higher material efficiency, superior accuracy, and resolution, making them well suited for intricate designs. However, these processes may incur higher costs and slower manufacturing speeds. The impact of sustainability is also crucial, although not measured in this paper, with other studies providing evidence of WAAM’s positive sustainability attributes [33,34].

The choice between WAAM and other AM methods depends on specific project requirements, considering factors such as part size (with the average size in the presented article being ideal for WAAM), low complexity in our case, necessary surface finish (requiring a study of wall geometry), and budget constraints, with WAAM proven to be a more cost-effective solution.

This work outlines the methodology for additive manufacturing using high deposition technology in titanium structures, covering the initial design stage, programming, process parameter definition, and concluding with an analysis of time, costs, and mechanical properties. This study represents a novelty in the field of additive manufacturing through the application of wire arc additive manufacturing (WAAM) technology to produce titanium aircraft components. The innovative aspect of this approach lies in the integration of a carefully conceived height compensation strategy, along with the incorporation of various deposition path techniques. From the thin-wall solution offered by single seam execution to the versatility provided by oscillation, each choice is a deliberate step toward demonstrating WAAM’s adaptability. With this nuanced approach, it is intended to contribute to the ongoing discourse in this field, opening avenues for further exploration and refinement in leveraging WAAM’s capabilities for aerospace applications.

## 2. Materials and Methods

### 2.1. Experimental Setup

**Figure 1:**
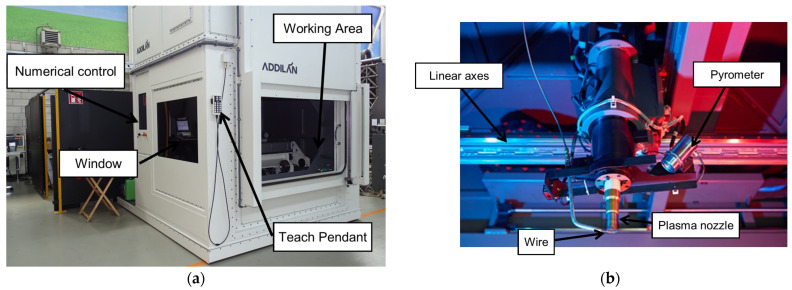
Experimental setup: (**a**) ADDILAN V0.1 from ADDILAN (Derio, Spain) machine equipped with (**b**) a plasma supply head.

The 3-axis Cartesian machine used in the manufacturing process of the component incorporates several essential components for its seamless operation. At the core of this setup is the Addilan V0.1 (ADDILAN, Derio, Spain), a versatile Cartesian machine that orchestrates precise movements and positioning throughout the manufacturing cycle. Supporting the welding process is the MagicWave5000+ PlasmaModule10 from Fronius (Wels, Austria), a piece of critical welding equipment that provides the necessary power to the PAW (Plasma Arc Welding) torch. This torch, identified as the PMW 350-2 from Autogen-Ritter (Feldkirchen-München, Germany), plays a pivotal role by generating the electric arc essential for the welding procedure. Additionally, the machine is equipped with various cameras for continuous monitoring and control. Notably, the MC500 Weld Camera by Redman (Wallingford, UK) allows for real-time observation of the welding process from outside the machine. Furthermore, the Tachyon 16k Camera from NIT (Madrid, Spain) provides valuable infrared imagery for analyzing the state of the molten pool, while the LIR320 Camera, also from NIT, offers insights into the thermal status of various machine components, the torch, and the part being manufactured. This comprehensive setup ensures precision and control in the manufacturing process. Figure 1 shows the plasma supply head equipped with the machine.

The ADDILAN v0.1 machine collects and stores all process monitoring data in a comma-separated values text file for further analysis. The main variables stored are the position of the axes (X, Y, Z), the process parameters (current, voltage, wire speed, advance speed), the state of the arc, and the environmental conditions of the machine (pressure, temperature, and oxygen).

The numerical control system developed by ADDILAN for this machine has been meticulously tailored to cater specifically to the WAAM (Wire Arc Additive Manufacturing) process, providing a set of distinct advantages that are currently unmatched in DED (Directed Energy Deposition) technologies. A standout feature of this system is the 3DLAN CAM software version v0.1, seamlessly integrated into the machine’s HMI (Human–Machine Interface). This innovative software streamlines the process by enabling the on-machine programming of parts, resulting in significant time savings by eliminating the need for extensive path programming.

Furthermore, the numerical control system fosters direct communication between the CAM software and the technological tables designed for specific technology–material combinations. This direct connection enhances precision and control over the manufacturing process, ensuring optimal outcomes. Additionally, the system facilitates direct communication between the CAM software and process sensors. This functionality enables the real-time adaptation of each layer of the additive manufacturing process to match the correct geometry, enhancing the overall quality and accuracy of the manufactured components.

**Figure 2:**
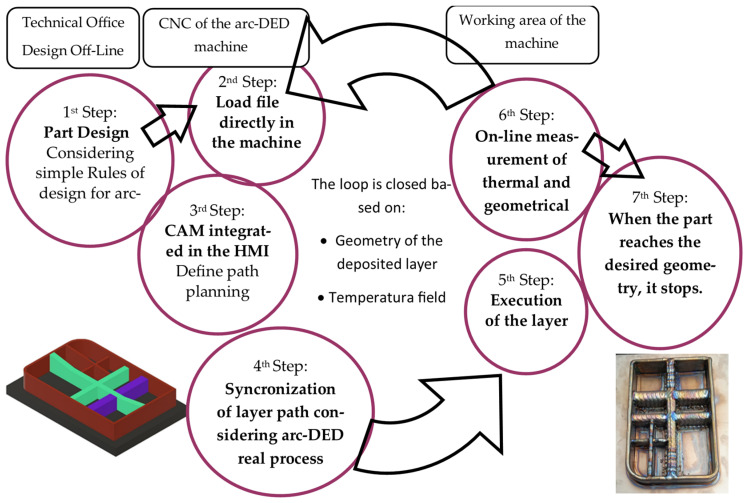
Diagram of CNC operation for preform manufacturing by wire arc-DED.

The phases for the manufacture of parts are shown schematically in Figure 2, considering the first stage of off-machine design carried out by the user of the technology. Once the part is designed, it is imported from the machine and all the programming is conducted from the HMI itself, as mentioned above. The machine’s numerical control has been specifically adapted to the WAAM process, featuring a closed-loop scheme specially adapted to Directed Energy Deposition (DED) technologies. The key features are to be found in everything that is done within machine control. By means of the CAM 3DLAN software, the machine’s integrated human–machine interface (HMI) paths from the off-machine design in Step 1 are programmed in Step 2. This software speeds up the programming of parts on the machine, saving path programming time. Direct communication occurs between CAM and the technology tables in Step 3, which facilitates the direct link between CAM and the technology tables developed for the material and technology, relating the deposition parameters to the correct bead generation and estimated layer geometry. Step 4 closes the direct communication between CAM and process sensors. It allows for the adaptation of each layer to the correct geometry by comparing the layer executed in Step 5 and the process sensors, including thermal sensors and the internal machine signals described for Step 6.

The schematic representation illustrates the manufacturing stages of the part, considering an initial off-machine design stage performed by the user of the technology. Once the part is designed, it is imported into the machine and all programming is executed directly from the HMI, as mentioned above.

### 2.2. Materials

As has been mentioned, the demonstrator was manufactured in grade 5 titanium (Ti_6_Al_4_V), provided in the form of a commercial wire with a 1.2 mm diameter from the company Böhler (Voestalpine, Linz, Austria). The composition of this thread is shown in Table 1.

A 20 mm thick flat titanium plate with dimensions of 360 × 270 mm^2^ was placed on the fixed table as a substrate. This substrate was sanded minutes prior to the manufacture of the part to eliminate oxidation. The composition of this substrate is the same as the filler material.

### 2.3. Geometry, Redesign, and Contribution Strategy

**Figure 3:**
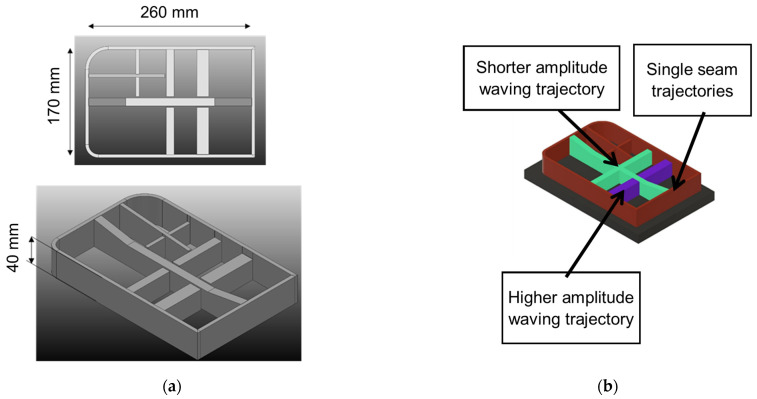
Titanium preforms to be manufactured using WAAM. (**a**) main dimensions of the part and (**b**) trajectories to print the part.

The aerospace industry is witnessing an increasing adoption of monolithic parts due to their numerous advantages. Figure 3 shows the part that is the subject of this paper. Monolithic parts, typically manufactured from a single piece of material, offer advantages such as reduced labor required during assembly and the minimal dependence on fasteners.

However, their adoption also presents specific challenges. Monolithic parts often have complex designs that integrate multiple functions into a single component. In addition, maintaining tight tolerances over long distances is crucial to their performance and safety.

To address these challenges and improve the precision of the machinery used in the production of aerospace monolithic parts, industry professionals are exploring innovative strategies and technologies. These include advances in design and manufacturing, the use of additive manufacturing techniques, and the implementation of automated quality control systems.

Ultimately, the goal is to manufacture monolithic parts with exceptional precision and consistency. This not only ensures the safety and reliability of aerospace systems, but also reduces production costs and labor requirements.

**Figure 4:**
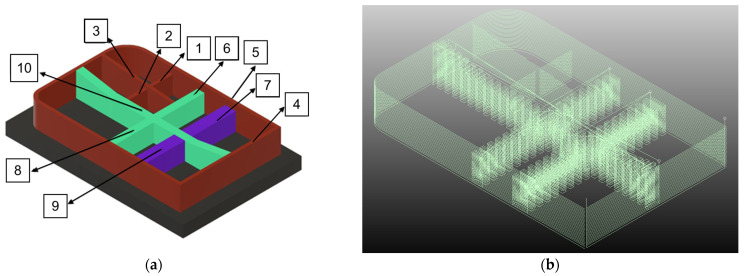
Numbering of the sub-parts of the original part: (**a**) discrete trajectories in order and (**b**) paths to print the part.

In the initial phase of manufacturing parts using PAW-based WAAM technology, an analysis of the original design was conducted to facilitate a swift and efficient production process through a redesign. Consideration was given to the junction points of crosses and their intersections with the outer contour. Consequently, a redesign of the part was deemed necessary to align with the manufacturing process. The spacing between trajectory paths plays a crucial role in achieving robust junctions. Therefore, the decision was made to segment the piece into 10 sections, as depicted in Figure 4a, with a particular emphasis on the joint distance.

The mono-seam deposition strategy in Wire Arc Additive Manufacturing (WAAM) offers precision and control over the welding path, leading to accurate layer-by-layer buildup and a potentially smoother surface finish. However, it may incur longer build times, particularly for large and complex components, as it follows a sequential layering process. On the other hand, the oscillating deposition strategy aims to achieve faster build times by covering larger areas in each layer and distributing heat more evenly, potentially reducing thermal distortion [35,36,37]. This approach may sacrifice some surface quality in intricate geometries and requires sophisticated programming for proper coordination. The choice between these strategies should consider the specific geometry of the aerospace component, desired material properties, and the need for optimal build speed and surface finish, emphasizing the importance of ongoing process optimization.

The part is set to be manufactured on a flat substrate with a thickness of 20 mm. The diverse geometries employed are illustrated in Figure 4, categorizing the initial five sections as Monoseam Geometry (labeled 1 to 5) and the subsequent five sections as Oscillatory Geometry (labeled 6 to 10).

Following the validation of the redesigned part, it is imported into the ADDILAN machine in Standard Triangle Language (STL format) to initiate the toolpath programming process. The selection of trajectory parameters, such as layer height and contribution strategies (monoseam and zig-zag), is imperative in this phase.

ADDILAN’s proprietary software enables precise positioning of the part, accounting for equipment characteristics. Trajectories are directly generated through the 3DLAN software on the ADDILAN v0.1 machine. These trajectories are determined based on past experiences in the manufacturing of analogous components. Achieving geometric tolerances is prioritized through the implementation of trajectories in opposite directions for consecutive layers. As previously outlined, the segmentation results in 10 distinct sections, each featuring unique trajectories.

### 2.4. Process Parameters

The welding mode that has been used in this production process is based on Plasma technology using a transferred arc and a MagicWave5000 generator together with the PlasmaModule10 plasma module, both obtained from the Fronius brand.

Due to the material used and the manufacturer’s recommendations, the protection gas used was pure argon (100% Ar) with a flow rate of 20 L/min, which will also act as a maintenance flow rate to maintain the optimal supply conditions in the machine. At the same time, the plasma gas flow rate was 1.2 L/min. The welding nozzle hole diameter used was 4 mm, and the electrode diameter was also 4 mm. The wire feeding speed was set at 4.3 m/min (1.3 kg/h).

To maximize productivity, waiting times between beads during manufacturing have been minimized. Temperature measurements were taken following the deposition of each seam, with a pyrometer strategically positioned at the finishing point of each seam providing accurate readings of the final material deposition temperature. A stringent control criterion was established, delaying the initiation of the subsequent seam or layer until the temperature attained a consistent 700 °C. In addition, the highest possible contribution rates are desired with an adequate wetting angle (around 30°) and a determined wall width, but without excessive thermal input and with adequate metallurgical integrity; the set of parameters can be seen in Table 2. The table comprises variables such as Strategy, Layer Number, Wire Feed Rate (WFR) in meters per minute, Ratio of Deposition (RD) in kilograms per hour, Current (I) in Amperes, Travel Speed (TS) in millimeters per second, and the estimation of the seam geometry, including Layer Width and Layer Height in millimeters. The variable Am represents the amplitude of the movement in the weaving (oscillatory path).

In this part, two types of deposition strategies are differentiated: the mono-seam strategy and the oscillatory strategy. Both strategies will follow the medial axis of the wall, but in the second, it will be combined with a weaving movement in the perpendicular direction. Therefore, the mono-seam strategy will be used for thin walls and the oscillatory strategy for thicker walls.

In the same way, and to keep the layer growth constant throughout the piece, in the shorter thin walls (crossing mono-seams), some parameters will be used where the thermal input is even lower than in the rest. On the other hand, in the first layers, the parameters were different until manufacturing stabilized (from the third layer). The initial layers in both strategies were executed with higher energy levels compared to the subsequent layers, as explained in [38]. The substrate material was cold at the beginning of the deposition process; therefore, higher energy levels were necessary during this initial layer to ensure proper material dilution and seam geometry. Regarding the inclination of the torch with respect to the substrate, it was worked with an angle of 90°, and the distance used between the welding nozzle and the substrate was 10 mm.

## 3. Results and Discussion

### 3.1. Manufacturing

**Figure 5:**
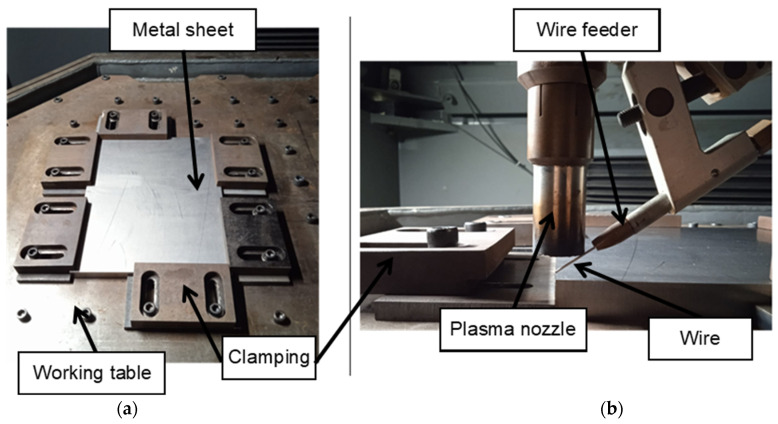
(**a**) Substrate placement in machine and (**b**) torch–substrate adjustment.

The manufacturing of the piece was carried out in the following way. The first step was to place the substrate on the machine table in a suitable position. Secondly, the substrate was tied using steel tools, thus fixing it in its position, as can be seen in Figure 5.

Prior to manufacturing and in order not to contaminate the contribution with any other substance (paint, grease, etc.), the upper surface of the substrate was cleaned with acetone. Next, the titanium spool was mounted in the machine, the rollers were placed, the wire was passed through them, and it was made to reach the end of the feed system of the torch. In this way, the tip of the wire was located vertically to the hole of the welding nozzle (or the tip of the electrode), and with that distance, the zeros of the substrate were searched manually. To do this, the tip of the wire was placed in the corner of the substrate that was taken as a reference for programming and the positions of the X and Y axes were noted. To search for zero on the Z axis, the tip of the wire touched the substrate and then moved 2 mm away (distance at which an optimum contribution has been approved). The supply chamber was inertized by emptying and filling cycles with inert gas (argon). Then, once a reduced oxygen concentration was obtained (less than 2000 ppm), the welding process began, adding the beads in consecutive layers.

**Figure 6:**
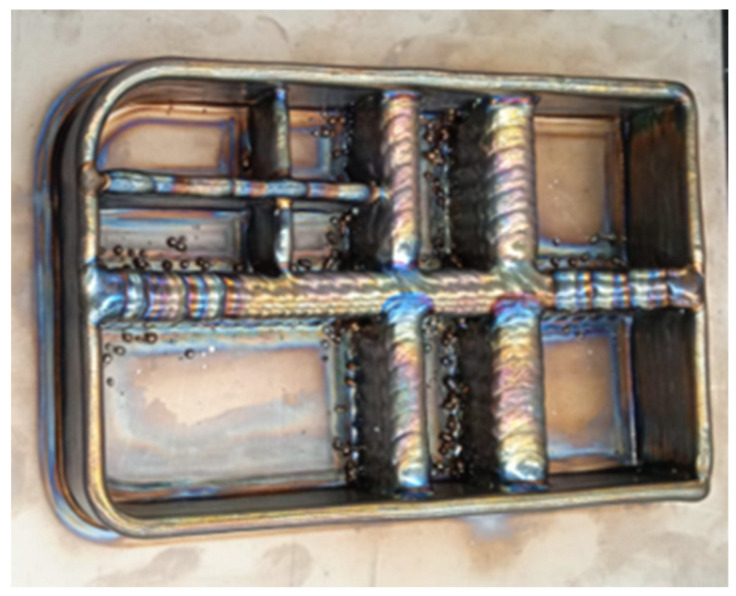
Photograph of the real titanium demonstrator in preform format after arc-DED printing.

The piece finally achieves dimensions of 260 × 170 × 45 mm. The wall width obtained in this piece is variable. Those contributed by the mono-seam strategy have a thickness of 8 mm. On the other hand, those carried out with the oscillation strategy have obtained a thickness that is the sum of the oscillation amplitude, and the 6 mm thickness of the seam that would be achieved with these parameters in the mono-seam strategy. That is to say, the seams that have an oscillation amplitude of 12 mm obtain a total thickness of 18 mm, and those with an amplitude of 18 mm have a thickness of 24 mm. Next, Figure 6 shows a photo of the manufactured part. The part manufactured is a preform, so the result of the part after WAAM additive manufacturing does not possess the surface quality typical of a final component. This phase precedes machining, serving as a preliminary step that replaces traditional forging, resembling an in situ forging close to the near-net shape (NNS) of the final part.

A small spatter on the oscillating walls can be seen on the part. In an oscillatory motion, the presence of spatter could be attributed to singular points where the direction changes, potentially causing issues with the proper flow of shielding gas. These points of directional change might disrupt the consistent and effective delivery of the protective gas, leading to spatter formation. To address this, it is crucial to optimize the gas flow and distribution to ensure uniform coverage across all directions of the oscillatory path. Additionally, adjusting the welding parameters, such as current and travel speed, can contribute to a more stable and controlled process, reducing the likelihood of spatter formation during WAAM with PAW.

Considering the geometry seen in Figure 6, it is suggested that post-Wire Arc Additive Manufacturing (WAAM) machining may be necessary to achieve the final geometry of the components. Additionally, there is an option to enhance the mechanical properties through heat treatment, as explored in [38]. While not mandatory, this heat treatment process, detailed in the referenced study, can be considered to further improve the mechanical characteristics of the manufactured components. The decision to undertake these additional steps would depend on the specific requirements and desired outcomes for the final aeronautical parts.

### 3.2. Monitoring

**Figure 7:**
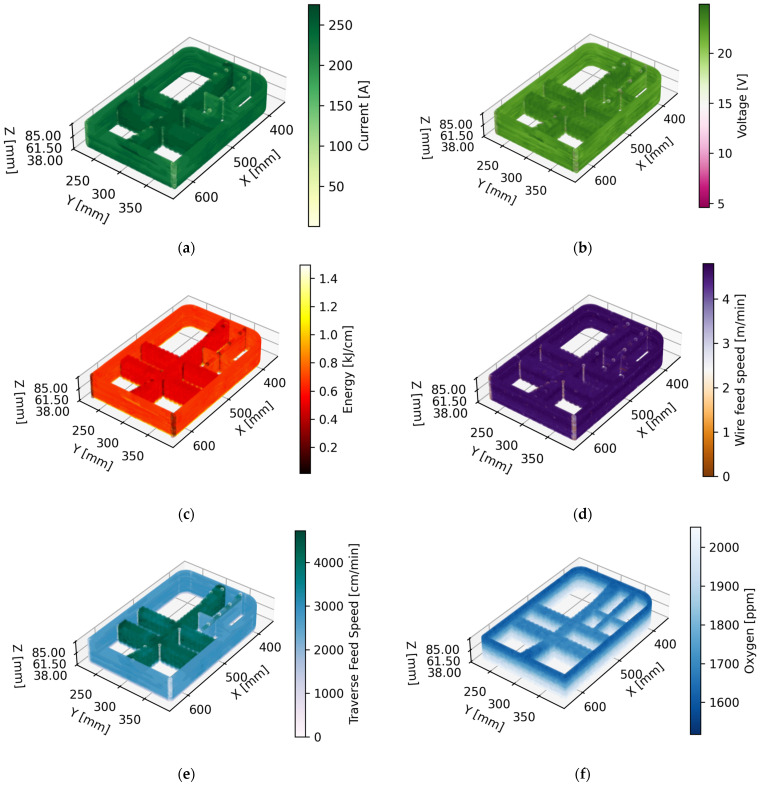
Main monitored process variables. From left to right and up–down: (**a**) Intensity, (**b**) Voltage, (**c**) Energy, (**d**) Wire feed speed, (**e**) Traverse feed speed, (**f**) ppm Oxygen level.

To facilitate a comprehensive investigation and derive insights conducive to enhancing future technology applications, a rigorous monitoring regime of welding parameters has been executed. The data acquisition process was configured to operate at a temporal resolution of 0.1 s, with data points meticulously recorded in the format of comma-separated values (CSVs). It is noteworthy that real-time graphical representations can be accessed at an impressively high update rate of 10 milliseconds. Nevertheless, to ensure signal fidelity and interpretability, a filtering mechanism has been instituted. This filter algorithm adeptly computes the rolling average based on a window size of 15 consecutive data points, effectively smoothing the signal for analytical purposes. Figure 7 illustrates the primary monitored process variables. This visual aid serves as a valuable reference for comprehending the dynamics of the welding process.

In Figure 7, a comprehensive view of the core monitored process variables essential for understanding the intricacies of the study is presented. From left to right and top to bottom, the variables include:

(a) Intensity: Reflecting the power input during welding.
(b) Voltage: Indicating the electrical potential applied during the process.
(c) Energy: Demonstrating the overall energy expenditure.
(d) Wire Feed Speed: The rate at which consumable wire is fed into the welding process.
(e) Traverse Feed Speed: Signifying the speed at which the welding head traverses along the workpiece.
(f) ppm Oxygen Level: The concentration of oxygen in parts per million (ppm) throughout the welding process.

It is important to note that this visual representation unveils distinctive trends. Notably, differences in energy consumption emerge when employing different welding strategies, ranging from oscillatory patterns characterized by higher speed and lower energy to mono-seam zones. Additionally, the oxygen level exhibits intriguing patterns, with lower ppm values observed at the outset of the process. These observations open avenues for deeper exploration and the potential optimization of welding strategies for enhanced outcomes in advanced manufacturing. Figure 7e represents the instantaneous velocity, capturing dynamic changes in motion, while Table 2 provides the velocity in the feed direction. In the context of the waving strategy, this feed direction velocity corresponds to the resultant velocity. Therefore, apparent differences arise due to the distinct representations of instantaneous and resultant velocities. This nuanced distinction contributes valuable insights into the Wire Arc Additive Manufacturing (WAAM) process, highlighting both the dynamic nature of the motion and the overall resultant effects in the feed direction. Clarifying this distinction enriches the understanding of the interplay between processing parameters and the dynamic behavior of the WAAM system.

### 3.3. Manufacturing Time

The manufacturing time was calculated based on various signals from the machine, outlined in Figure 7, including the time calculated from internal machine signals, the time taken for filling indicated by the vacuum chamber signal, and the waiting time, which is the time between two points of active torch movement (active torch defined as having positive current and in motion), representing the printing time. Finally, the remaining time until the completion of the piece is considered to be the preparation time.

**Figure 8:**
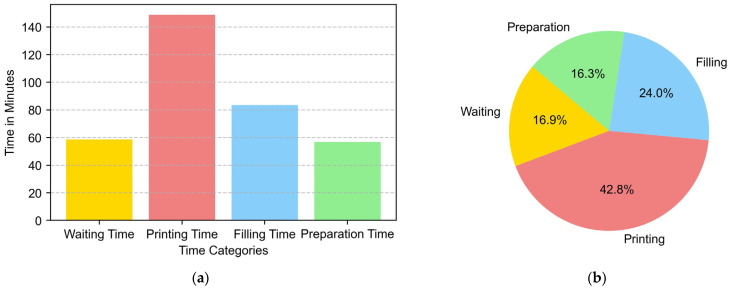
Manufacturing times: (**a**) time breakdown in minutes and (**b**) time comparison (percentages of the manufacturing time).

The objective of additive manufacturing is to provide a competitive alternative to traditional manufacturing methods. For this reason, manufacturing time is a relevant parameter when planning the process. The results are shown in Figure 8. In the piece of this study, the manufacture was carried out in a single day, with the total time required being 6 h, of which 1 h was taken to prepare the machine, 1 h and a half for filling the machine, and 3 and a half hours of manufacturing. The total contribution time was 2 h and 30 min.

In this case, the total time needed considering the input, stops due to failures, and other aspects was 3:30 h. The manufacturing time breakdown is shown in Figure 8 above. It was noy necessary to change the gas cylinder since we connected directly to a block of Argon cylinders. Neither were any coil changes made since the weight of the coil (10 kg) was greater than the weight of the piece.

### 3.4. Economical Study

**Figure 9:**
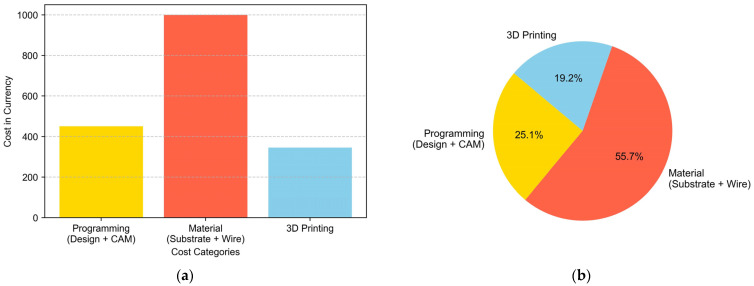
Manufacturing costs: (**a**) cost breakdown in euros and (**b**) cost comparison (percentages of the manufacturing costs).

Below is the cost breakdown made for the manufacturing of the target part. Within the printing cost, all expenses related to machine amortization, chamber filling protection gases, and torch consumables have been considered. As can be seen in Figure 9, the material used accounts for 56% of the manufacturing cost, which is logical in the case of a material like titanium.

The budget allocation of EUR 450 for programming (design + CAM) reflects the critical role of skilled labor and software tools in the manufacturing process. Ongoing scrutiny is needed to ensure that costs align with the complexity of the design tasks and evolving software requirements. Allocating EUR 1000 for materials (substrate + wire) emphasizes the importance of quality inputs. Evaluating the cost-effectiveness of chosen materials requires considering their role in the final product and exploring alternative, cost-efficient options on the market. The budget of EUR 345 for 3D printing underscores the significance of this technology. For 3D printing costs, the energy consumed during the printing process is an important factor, as is the total printing time, considering an hourly rate based on the operator costs associated with supervision or human intervention during printing. The cost of consumables must also be considered. Machine maintenance and depreciation costs have been left out of the calculation. In summary, there is a need for more comprehensive economic and sustainability evaluations in real-world applications given the evolving nature of technology.

### 3.5. Mechanical Characterization

The mechanical characterization of the Ti6Al4V preforms produced via Wire Arc Additive Manufacturing (WAAM) technology involved a meticulous assessment of tensile properties following a thermal treatment at 720 °C for 2 h and 20 min. The tensile tests were conducted at room temperature using an Istron 5585 H electronic machine equipped with a contact extensometer and a 100 kN load cell. The testing procedure adhered to the guidelines outlined in ISO 6892-1:2019 [39], specifically focusing on the method of conducting tensile tests at room temperature. The manufacturing properties of the tested wall are those of the long mono-seam wall in the upper layers as the lower layers are discarded for the extraction of the specimens. The difference between the mechanical values in oscillation and mono-seam properties would be of interest for future work.

**Figure 10:**
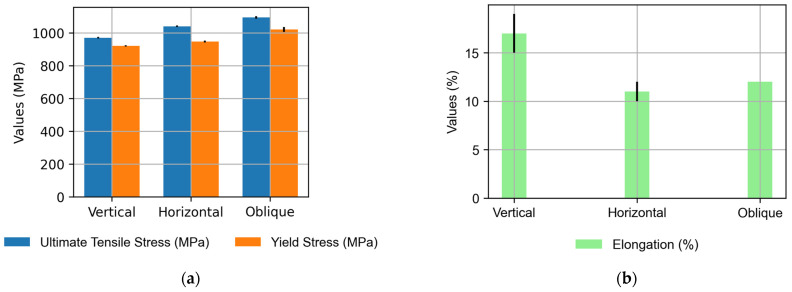
Mechanical properties of Ti_6_Al_4_V manufactured by arc-DED in the three test directions: (**a**) UTS and YS and (**b**) elongation.

The tensile testing setup comprised three probes in the horizontal direction and six in the vertical, as illustrated in Figure 10. The results, as summarized in Table 3, revealed a favorable overall performance, but nuances in mechanical behavior were evident across different orientations.

The tensile testing outcomes demonstrate the robustness of the Ti_6_Al_4_V preforms. Vertical specimens exhibited an average ultimate tensile stress of 970 MPa and an average yield stress of 920 MPa, which are well within the prescribed range. However, the horizontal specimens displayed interesting variations. While the average ultimate tensile stress was commendable at 1040 MPa, the elongation and reduced area percentages fell short of expectations.

The observed variations in the mechanical properties hint at the anisotropic nature inherent in WAAM-fabricated components. The vertical specimens, representing the build direction, demonstrated consistent and expected performance. In contrast, the horizontal specimens exhibited lower elongation and reduced area percentages, indicative of a potential susceptibility to fracture under tensile loading.

Anisotropic behavior poses implications for the application of these components, especially in scenarios where tensile ductility is a critical factor. Future efforts should delve into refining process parameters and post-processing techniques to enhance the uniformity of mechanical properties across different orientations.

The mechanical characterization of annealed WAAM Ti6Al4V preforms highlights their overall strength and resilience. The observed variations, particularly in the horizontal specimens, point towards opportunities for optimization. This study contributes valuable insights to the ongoing efforts in refining WAAM processes for Ti_6_Al_4_V components, with a focus on achieving consistent mechanical performance across various orientations, crucial for applications in aerospace and high-performance industries.

## 4. Conclusions

In the present work, the methodology followed for the manufacture of high-deposition metal parts is explained. In a synthesized way, the main conclusions are:

- The methodology followed for the redesign of parts using WAAM technology has been explained, being necessary to separate the geometry in different sections considering the geometric dimensions and the intersections between them.
- Each section has been assigned a predefined trajectory, considering the possibility of manufacturing different strategies, such as single seam trajectories, overlapping, or oscillation. The operation of the control that facilitates the use of the machine has been explained, combining the knowledge of each material with the associated manufacturing strategy.
- The impact has been shown both on manufacturing times and on the cost of the same to understand the importance of each variable. The breakdown revealed that 1 h was allocated for machine preparation, 1.5 h for filling the machine, and 3.5 h for actual manufacturing, with a total contribution time of 2 h and 30 min. The economic study highlights a comprehensive cost breakdown for manufacturing the target part, with material costs constituting 56% of the total.
- The tensile testing results underscore the robustness of Ti6Al4V preforms, with vertical specimens exhibiting commendable mechanical properties, including an average ultimate tensile stress of 970 MPa and an average yield stress of 920 MPa, well within the specified range. Notably, the horizontal specimens demonstrated a noteworthy ultimate tensile stress of 1040 MPa; however, their elongation and reduced area percentages fell below expectations, suggesting potential susceptibility to fracture under tensile loading. These variations highlight the anisotropic nature inherent in Wire Arc Additive Manufacturing (WAAM)-fabricated components, emphasizing the importance of optimizing build orientations to ensure consistent and reliable mechanical performance in critical applications.
- Future directions for this work include delving into the sustainability analysis of the process as an alternative to conventional mechanical manufacturing for operational components. From a process perspective, potential avenues involve enhancing productivity, optimizing the mechanical characteristics of the material, or refining the resemblance to the final target shape.

## Figures and Tables

**Table 1:** Composition of titanium welding wire.

Material	C	Fe	N	H	O	Al	V	Ti
Ti_6_Al_4_V	<0.08	<0.25	<0.05	<0.015	<0.2	5.5–6.8	3.5–4.5	rest.

**Table 2:** Manufacturing parameters of the part.

Strategy	Layer Number	WFR [m/min]	RD [kg/h]	I [A]	TS [mm/s]	Layer Width [mm]	Layer Height [mm]
Single seam (short)	1	2.7	0.8	150	3.8	8	23
	2	2.7	0.8	150	5.7	8	1.3
	3	2.7	0.8	150	7.6	8	1.3
Single seam (long)	1	4.3	1.3	225	3.8	8	23
	2	4.3	1.3	225	5.7	8	1.3
	Rest	4.3	1.3	225	7.6	8	1.3
Oscillatory (Am = Amplitude of oscillation)	1	4.3	1.3	250	6	6 + Am	2.4
	2	4.3	1.3	250	9	6 + Am	1.4
	Rest	4.3	1.3	250	12	6 + Am	1.4

**Table 3:** Mechanical results in the three tested directions in a tensile test.

Direction	Ultimate Tensile Stress (MPa)	Yield Stress (MPa)	Elongation (%)
Vertical	970 ± 5	920 ± 4	17 ± 2
Horizontal	1040 ± 4	947 ± 6	11 ± 1
Oblique	1094 ± 7	1020 ± 17	12 ± 0

## Data Availability

Data will be available on request to the corresponding author.
